# Establishment and validation of a novel rat model of early-stage patellofemoral osteoarthritis induced by patellar instability

**DOI:** 10.3389/fsurg.2026.1876653

**Published:** 2026-09-08

**Authors:** Fangli Yang, Zhen Wang, Mengmeng Li, Mingwei Shi, Jieyao Diao, Xuanqiang Fan, Tao Yang, Yunfeng Zhou, Hui Xu

**Affiliations:** 1 Henan University of Chinese Medicine, Zhengzhou, China; 2 Department of Rehabilitation Medicine, Jiaozuo People's Hospital, Jiaozuo, China; 3 Third Affiliated Hospital of Henan University of Traditional Chinese Medicine, Zhengzhou, China; 4 Guangdong Vocational Institute of Public Administration, Guangzhou, China

**Keywords:** animal model, joint compartment specificity, osteoarthritis, patellar instability, patellofemoral osteoarthritis

## Abstract

**Objective:**

This study aimed to establish and validate a rat model of patellofemoral osteoarthritis (PFOA) induced by patellar instability and to characterize its early degenerative features and compartment specificity.

**Methods:**

Twenty male Sprague-Dawley rats were assigned to four groups (*n* = 5 per group): a control group and PFOA model groups evaluated at 1, 2, and 3 weeks after surgery. PFOA was induced in the left knee by transecting the medial one-third of the patellar ligament and medial patellar retinaculum, after which the patella was gently displaced laterally. With the patella maintained in a mildly lateralized position, the lateral peripatellar soft tissues were sutured at intervals and secured to the lateral joint capsule. Cartilage degeneration was assessed by hematoxylin and eosin staining and Mankin scoring. Cartilage matrix changes were evaluated using Safranin O-Fast Green and toluidine blue staining. Synovial lavage fluid and serum levels of procollagen II C-terminal propeptide (PIICP), matrix metalloproteinase-13 (MMP-13), C-terminal telopeptide of type II collagen (CTX-II), and cartilage oligomeric matrix protein (COMP) were measured by ELISA.

**Results:**

At 1 week after surgery, patellofemoral cartilage showed surface irregularity, disrupted zonal architecture, and abnormal chondrocyte arrangement, with progressive worsening over time. In contrast, tibiofemoral changes were milder and appeared later. Safranin O-Fast Green and toluidine blue staining showed earlier and more pronounced loss of proteoglycans and glycosaminoglycans in patellofemoral cartilage than in tibiofemoral cartilage, accompanied by an earlier reduction in positively stained area. Compared with controls, PIICP levels in synovial lavage fluid and serum decreased at all postoperative time points, whereas MMP-13, CTX-II, and COMP levels increased (*P* < 0.05). These changes were more pronounced at 2 and 3 weeks than at 1 week, with no significant difference between 2 and 3 weeks, suggesting that the model reached a relatively stable early degenerative phase by approximately 2 weeks after surgery.

**Conclusion:**

Combined partial transection of the patellar ligament and medial patellar retinaculum with lateralized patellar tracking successfully established an early-stage rat PFOA model with preferential patellofemoral involvement. This model rapidly induced cartilage metabolic imbalance and histological degeneration, showing compartment specificity and potential value for studying early PFOA pathogenesis and interventions.

## Introduction

Knee osteoarthritis (KOA) is a common degenerative joint disease affecting middle-aged and older adults. Its major pathological features include articular cartilage degeneration, subchondral bone remodeling, synovial inflammation, and osteophyte formation (1, 2). Based on the predominant compartment involved, KOA can be classified into tibiofemoral osteoarthritis (TFOA), patellofemoral osteoarthritis (PFOA), or combined TFOA and PFOA (3). Epidemiological studies have shown that PFOA is common. The prevalence of PFOA is approximately 23.9% in the general population and may reach 39% among individuals with knee pain. In some patients, PFOA presents as with isolated patellofemoral involvement; however, compared with TFOA, PFOA has received relatively limited attention (4, 5).

It is generally accepted that the development and progression of PFOA are closely associated with abnormal patellar tracking, joint malalignment, and altered distribution of local mechanical stress (6). The stability of the patellofemoral joint (PFJ) is maintained by the coordinated effects of osseous structures, the capsuloligamentous complex, and the quadriceps muscle. The medial patellofemoral ligament and associated medial retinacular structures play important roles in limiting lateral patellar displacement (7, 8). When these medial stabilizing structures are compromised, the patella becomes more susceptible to lateral displacement, lateral tilt, or a tendency toward subluxation. These changes may reduce the PFJ contact area, increase focal stress concentration, and alter joint kinematics. Consequently, they may induce cartilage matrix, phenotypic alterations in chondrocytes, and subchondral bone remodeling, ultimately leading to pathological changes characteristic of PFOA. Existing animal studies have also demonstrated that interventions targeting patellar position or stability can induce degeneration of PFJ cartilage and subchondral bone. Therefore, the development of a PFOA animal model based on patellar instability has a clear pathobiological and biomechanical rationale (9).

Existing animal models of PFOA, including spontaneous, surgically induced, chemically induced, and mechanical overload models, all have inherent limitations. Spontaneous models are limited by a prolonged disease course and considerable individual variability. Classic surgical models, such as anterior cruciate ligament transection and meniscal injury, primarily affect the tibiofemoral joint (TFJ), with patellofemoral changes largely developing as secondary lesions. Chemically induced models, such as intra-articular injection of monosodium iodoacetate (MIA), can rapidly induce lesions but mainly reflect a chemically driven injury process. Mechanical overload models are difficult to standardize and lack compartment specificity. Therefore, establishing a relatively straightforward animal model that simulates early patellofemoral joint degeneration associated with patellar instability and shows a degree of compartment specificity remains valuable for research.

Early degeneration in PFOA involves not only structural cartilage damage but also loss of matrix components and imbalance in cartilage metabolism (10). Reductions in proteoglycans and glycosaminoglycans indicate impaired cartilage matrix homeostasis. Moreover, biomarkers such as procollagen II C-terminal propeptide (PIICP), matrix metalloproteinase-13 (MMP-13), C-terminal telopeptide of type II collagen (CTX-II), and cartilage oligomeric matrix protein (COMP) provide distinct perspectives on type II collagen synthesis, matrix degradation, cartilage injury, and remodeling. Based on this rationale, the present study aimed to establish a rat model of PFOA by transecting the medial one-third of both the left patellar ligament and the medial patellar retinaculum, followed by lateral mechanical impaction of the patella to induce lateral displacement or a tendency toward subluxation. The early degenerative characteristics and compartment specificity of the model were subsequently evaluated at multiple levels—including cartilage structure, matrix composition, and metabolic status—using histological staining, matrix component analysis, and enzyme-linked immunosorbent assay (ELISA).

### Characteristics of existing animal models of patellofemoral osteoarthritis

#### Spontaneous models of patellofemoral osteoarthritis

Spontaneous PFOA models refer to models in which animals gradually develop degenerative changes in the PFJ during aging, without external intervention. The main advantage of these models lies in their ability to reflect, to a certain extent, the natural progression of the disease, with minimal interference from surgical or chemical stimuli. Representative spontaneous models include Hartley guinea pigs and STR/ort mice, which may exhibit PFJ cartilage degeneration and associated bone structural abnormalities at specific ages. Studies have shown that spontaneous PFJ cartilage degeneration and subchondral bone microstructural damage can occur in Hartley guinea pigs aged 3–5 months. In STR/ort mice, osteoarthritis-like changes in the PFJ may even be detected earlier than those in the TFJ (11, 12).

However, spontaneous models are characterized by a long disease course and considerable individual variability. Moreover, the degenerative process is often not confined to the PFJ and is frequently accompanied by TFJ degeneration. Consequently, it is difficult to precisely determine the time of onset of patellofemoral lesions and their temporal relationship with tibiofemoral lesions. Therefore, spontaneous models are more suitable for studies of aging-related or long-term natural disease progression, but they are not the preferred models for mechanistic studies specific to the PFJ.

#### Surgically induced patellofemoral osteoarthritis models

Surgically induced models produce joint degeneration within a relatively short period by disrupting joint-stabilizing structures or altering the local mechanical environment. These models are characterized by relatively short induction periods and ease of implementation. In previous studies, degenerative changes in PFJ cartilage and subchondral bone have been observed following anterior cruciate ligament injury or transection. Meniscal displacement or meniscectomy can also induce PFOA-like lesions associated with synovitis and abnormal gait kinematics (13–15). These findings suggest that although classic knee surgery-based models are not specifically established to target the PFJ, they can produce evident patellofemoral involvement. Furthermore, some researchers have attempted to establish models involving abnormalities of patellar position or stability. For instance, patella baja models can lead to degeneration of PFJ cartilage and subchondral bone, while models involving reduced patellar loading during growth or patellar instability can induce femoral trochlear dysplasia and early patellofemoral cartilage degeneration (16, 17). Among these models, the patellar instability model has been used to investigate the relationship between the activation of NF-κB and JAK1/STAT3 signaling pathways and cartilage changes, demonstrating its utility in mechanistic studies (18, 19).

Overall, surgically induced models represent a relatively controllable approach to establishing PFOA. However, most surgical models, such as anterior cruciate ligament transection and meniscal injury, primarily target the TFJ. Patellofemoral lesions are often secondary to or concomitant with global alterations in gait or whole-knee inflammatory responses. Although some researchers have developed surgical procedures more closely related to patellofemoral mechanics, such as the induction of patella baja or patellar instability, variations persist in the degree of lateral displacement, fixation methods, the presence or absence of complete dislocation, and postoperative gait compensation. These variations affect model standardization and compartment specificity. An optimized surgically induced PFOA model should therefore use a clearly defined anatomical intervention, maintain a controllable degree of patellar maltracking, and minimize direct disruption of the primary stabilizing structures of the TFJ. A key challenge in further refining surgical models is to induce earlier and more localized pathological changes in the PFJ. Meanwhile, the structure and function of the TFJ should be largely preserved.

#### Chemically induced models of patellofemoral osteoarthritis

Chemically induced models rapidly induce cartilage damage by injecting metabolic inhibitors, such as MIA, into the joint cavity. These process cause chondrocyte metabolic dysfunction and cell death, resulting in osteoarthritis-like changes, including cartilage matrix loss, cartilage fissuring, and synovialinflammation. These models are characterized by a short induction period and high reproducibility. The severity of the resulting lesions can be controlled by adjusting the dose administered. Moreover, they can produce cartilage and synovial pathological changes across different stages of disease, including cartilage degeneration, osteophyte formation, and synovitis. Takahashi et al. established an experimental rat model of PFOA through local injection of MIA into the PFJ, demonstrating that chemical induction can induce well-defined PFJ lesions within a relatively short period (20).

However, chemically induced models are based on chemically cytotoxic cytotoxic injury, which differs from the chronic degeneration caused by abnormal patellar tracking or local mechanical imbalance. Therefore, these models do not fully replicate the natural progression of clinical PFOA. Although these models can rapidly induce lesions and allow the severity of these lesions to be controlled, their patellofemoral specificity relies primarily on precise injection localization than the reproduction of biomechanical abnormalities associated with patellar instability. Consequently, these models are more suitable for investigating mechanisms underlying cartilage damage and acute inflammatory responses than for reproducing the overall disease course of PFOA associated with patellar instability.

#### Mechanical load-induced models patellofemoral osteoarthritis

An abnormal mechanical environment can disrupt intra-articular homeostasis and thereby induce PFOA. Clinically, abnormal patellar tracking, trochlear dysplasia, and altered lower limb alignment can all lead to an abnormal distribution of contact stress within the PFJ, resulting in cartilage wear, inflammatory responses, and accelerated cartilage degeneration. Mechanical load-induced models simulate joint degeneration resulting from mechanical imbalance by applying excessive mechanical loads or removing normal physiological loading. A previous review indicated that mechanical loading plays a crucial regulatory role in maintaining articular cartilage homeostasis. The application of supraphysiological loads to the PFJ through dynamic flexion-extension or compression can affect cartilage structure and metabolism, leading to mechanical damage to chondrocytes and matrix degradation (21). In animal experiments, hindlimb suspension has been shown to cause abnormal knee alignment, lateral patellar displacement, and PFJ degeneration in growing rats (22). Conversely, a partial reduction in weight bearing in MIA-induced PFOA models can alleviate cartilage degeneration, suggesting that the mechanical environment exerts a bidirectional influence on the progression of PFOA (23).

Although such models offer certain advantages for elucidating the mechanical mechanisms underlying PFOA, they require specialized equipment and involve complex parameter settings, making them difficult to standardize. Furthermore, mechanical loading interventions often simultaneously alter the weight-bearing and gait patterns of the entire knee joint, making it difficult to confine pathological changes specifically to the PFJ. Therefore, these models are more suitable for investigating the relationship between mechanical abnormalities and joint degeneration, but they still have limitations in achieving PFJ-specific modeling.

In summary, existing animal models of PFOA have distinct advantages and limitations. However, some models primarily involve the entire knee joint or the TFJ, with patellofemoral lesions appearing mainly as secondary changes. Models that simultaneously reproduce localized mechanical imbalance and preferential PFJ involvement remain limited. To address this gap, the present study employed a modeling strategy centered on weakening the medial stabilizing structures to induce lateral patellar displacement. Specifically, the medial one-third of both the patellar ligament and medial patellar retinaculum of the left knee were transected, creating a tendency toward lateral patellar displacement or subluxation. Subsequently, the lateral peripatellar soft tissues were sutured at intervals and secured to the lateral joint capsule to maintain the patella in a mildly lateralized position. This procedure was intended to improve model reproducibility and compartment specificity. It may also better reflect the early pathological features of PFOA without directly disrupting the major stabilizing structures of the TFJ.

## Materials and methods

### Experimental animals and grouping

Twenty healthy 8-week-old male Sprague-Dawley rats of specific pathogen-free (SPF) grade, weighing 280 ± 20 g, were obtained from Vital River Laboratory Animal Technology Co., Ltd. (Production License No.: SYXK (Yu, Henan) 2021-0015). The animals were housed in the SPF facility of the Animal Experiment Center at Henan University of Chinese Medicine, with *ad libitum* access to food and water. The housing conditions were maintained at 21 ± 2 °C, a relative humidity of 40%–60%, and a 12-h light/12-h dark cycle. This study was conducted in accordance with relevant guidelines for laboratory animal ethics and welfare. The study protocol was approved by the Laboratory Animal Welfare Ethics Committee of Henan University of Chinese Medicine (approval no.: IACUC-202506006).

After a 1-week acclimatization period, the rats were randomly assigned using a random number table to four groups of five animals each: a control group and the 1-, 2-, and 3-week model groups. Rats in the control group underwent no surgical intervention and were maintained under the same housing conditions. They were euthanized and sampled at the same time point as the 3-week model group. In the three model groups, PFOA was induced in the left knee, and tissue samples were collected at 1, 2, or 3 weeks after surgery, according to group allocation.

### Establishment of the patellofemoral osteoarthritis model

Food was withheld for 12 h before surgery, whereas water remained available *ad libitum*. Anesthesia was induced by an intraperitoneal injection of 3% sodium pentobarbital at a dose of 30 mg/kg body weight. After the loss of the righting and withdrawal reflexes, the rats were placed and secured in a supine position on the operating table. The left hind limb was slightly abducted, with the knee maintained in extension. After routine shaving and disinfection of the left knee region, a longitudinal skin incision approximately 1.0–1.5 cm long was made along the medial border of the left patella. The skin, subcutaneous tissue, and superficial fascia were incised sequentially. The surrounding soft tissues were bluntly dissected to expose the patella, patellar ligament, and medial patellar retinaculum. Excessive soft-tissue retraction was avoided. The lateral patellar supporting structures, quadriceps tendon, remaining joint capsule, and major stabilizing structures of the TFJ were preserved.

The medial one-third of the medial patellar retinaculum, including the medial patellofemoral ligament, and the medial one-third of the patellar ligament were transected to compromise medial patellar stability. After the release, the patella was gently displaced laterally using non-toothed forceps. Successful model establishment was confirmed by a clear tendency toward lateral displacement or subluxation, together with lateral maltracking during knee flexion and extension. All observations were recorded by the same researcher. Subsequently, with the patella maintained in a mildly lateralized position, the lateral soft tissues were sutured at intervals and secured to the lateral joint capsule. This procedure was intended to maintain a lateralized patellar tracking tendency rather than to rigidly immobilize the patella. After suturing, passive knee flexion and extension were performed to confirm that the patella remained mobile and exhibited lateral maltracking without complete dislocation. After hemostasis was confirmed, the peripatellar soft tissues and skin were closed in layers.

### Specimen collection and preservation

Rats in the 1-, 2- and 3-week model groups were sacrificed at the corresponding time points,whereas rats in the control group were euthanized at the same time point as the 3-week model group. Under deep anesthesia, blood samples were collected from the abdominal aorta. The rats were subsequently euthanized via intraperitoneal administration of an overdose of 3% sodium pentobarbital (200 mg/kg body weight). Death was confirmed by the absence of respiration, heartbeat, corneal reflex, and response to a toe pinch. Synovial fluid and left knee joint specimens were then collected.

### Serum collection

Approximately 1 mL of blood was collected from the abdominal aorta of each rat. The blood samples were placed in sterile clot-activator tubes, allowed to stand at room temperature for 30 min, and then centrifuged at 3,000 rpm for 10 min. The separated serum was transferred to cryovials and stored at −80 °C until further analysis.

### Synovial lavage fluid collection

Synovial lavage fluid was collected from the left knee joint. After disinfection, the knee was slightly flexed to expose the suprapatellar bursa and PFJ region. A microsyringe was inserted into the joint cavity through a superolateral or lateral parapatellar approach. Then, 150 μL of sterile PBS was slowly injected into the left knee joint cavity. The knee was gently flexed and extended to allow adequate mixing of the lavage fluid within the joint space. Approximately 100 μL of lavage fluid was then carefully aspirated and transferred into a centrifuge tube. Repeated punctures and blood contamination were avoided; visibly bloodstained samples were discarded. The collected lavage fluid was centrifuged at 1,500 rpm at 4 °C for 5 min to remove debris and impurities. The supernatant was aliquoted and stored at −80 °C until further analysis. Because the same PBS lavage volume and collection procedure were used for all animals, the measured biomarker concentrations were compared under standardized dilution conditions.

### Knee joint tissue collection

After blood and synovial fluid collection, the entire left knee joint was harvested. Excess surrounding soft tissues was removed, while the integrity of the PFJ, TFJ, joint capsule, and associated bony structures was preserved as much as possible. Osteotomies were performed approximately 1–1.5 cm proximal to the femoral condyles and 1–1.5 cm distal to the tibial plateau to obtain an intact knee joint specimen. The specimens were then fixed in 4% paraformaldehyde for subsequent histological analyses. To allow separate evaluation of patellofemoral and tibiofemoral cartilage degeneration, the PFJ and TFJ regions were subsequently processed as separate anatomical regions during histological preparation. PFJ sections were used to assess the articular cartilage of the patella and femoral trochlea, whereas the TFJ sections were used to assess the articular cartilage of the femoral condyle and tibial plateau.

### Histological processing, staining, and evaluation

Fixed knee joint specimens were decalcified at room temperature in a 10% EDTA solution (pH 7.2–7.4), which was replaced every 24 h until adequate decalcification was achieved. After decalcification, the specimens were rinsed under running water, dehydrated through a graded ethanol series, cleared in xylene, and embedded in paraffin. Sagittal sections of 5 μm in thickness were prepared. Representative sagittal sections from the PFJ and TFJ were selected separately for compartment-specific histological staining, scoring, and quantitative image analysis. All sections were examined and imaged uning identical microscope settings. The animal was used as the statistical unit, and the mean values obtained from representative sections from each animal were used for statistical analysis.

Hematoxylin and eosin (H&E) staining was used to assess the overall histomorphological features of articular cartilage. Paraffin sections were routinely deparaffinized and rehydrated. After being stained with hematoxylin, the sections were differentiated and blued, and then counterstained with eosin. Tissue processing was completed through graded ethanol dehydration, xylene clearing, and mounting with neutral balsam. Cartilage surface integrity, layered structure, chondrocyte morphology and arrangement, and local architectural disorganization in the PFJ and TFJ were examined under a light microscope. Cartilage degeneration in the PFJ and TFJ was semiquantitatively evaluated using the Mankin scoring system (24). The scoring criteria included cartilage structure, chondrocyte abnormalities, matrix staining, and tidemark integrity, with higher total scores indicating more severe degeneration. All scoring was performed by investigators blinded to group allocation.

Histological changes in the cartilage matrix were evaluated using Safranin O–Fast Green and Toluidine Blue staining. Paraffin sections were routinely deparaffinized and rehydrated, followed by staining according to the manufacturer's instructions. After staining, the sections were dehydrated through a graded ethanol series, cleared in xylene, and mounted with neutral balsam. The sections were examined and image under a light microscope. Positively stained areas were quantified, and the percentage of positively stained area (%) was calculated. Safranin O–Fast Green staining was used to assess proteoglycan retention in the cartilage matrix and the extent of proteoglycan loss. Toluidine Blue staining was used to visualize the distribution of glycosaminoglycans (GAGs) and changes in matrix staining, reflecting alterations in GAG content. Image acquisition and analysis conditions were maintained consistently consistent across experimental groups to ensure comparability.

### ELISA

Frozen synovial fluid and serum samples were thawed at 4 °C and thoroughly mixed before analysis. The concentrations of PIICP, MMP-13, CTX-II, and COMP were measured using the respective ELISA kits according to the manufacturers’ instructions. A series of standards were prepared, and a standard curve was generated for each biomarker. Samples were appropriately diluted based on the results of preliminary experiments. Each sample was assayed in duplicate. All samples used to measure the same biomarker were processed in the same batch to minimize inter-assay variation. Biomarker concentrations were calculated using the corresponding standard curves.

### Statistical analysis

Statistical analysis were performed using SPSS version 26.0 (IBM Corp, Armonk, NY, USA), and graphs were generated with GraphPad Prism version 10.0 (GraphPad Software, San Diego, CA, USA). Continuous data were expressed as the mean ± standard deviation (mean ± SD. One-way analysis of variance was used for comparisons among multiple groups. When the overall difference was statistically significant, *post hoc* pairwise comparisons were performed using the Bonferroni correction. A two-sided *P* value < 0.05 was considered statistically significant.

## Results

All rats completed the experimental and sample collection procedures. All rats in the model groups successfully underwent surgery. During the early postoperative period, the model group showed mild swelling of the left knee and transient lameness. No incision-related infections, serious complications, or deaths were observed during the experiment.

### Cartilage degeneration occurred earlier in the PFJ than in the TFJ

**Figure 1:**
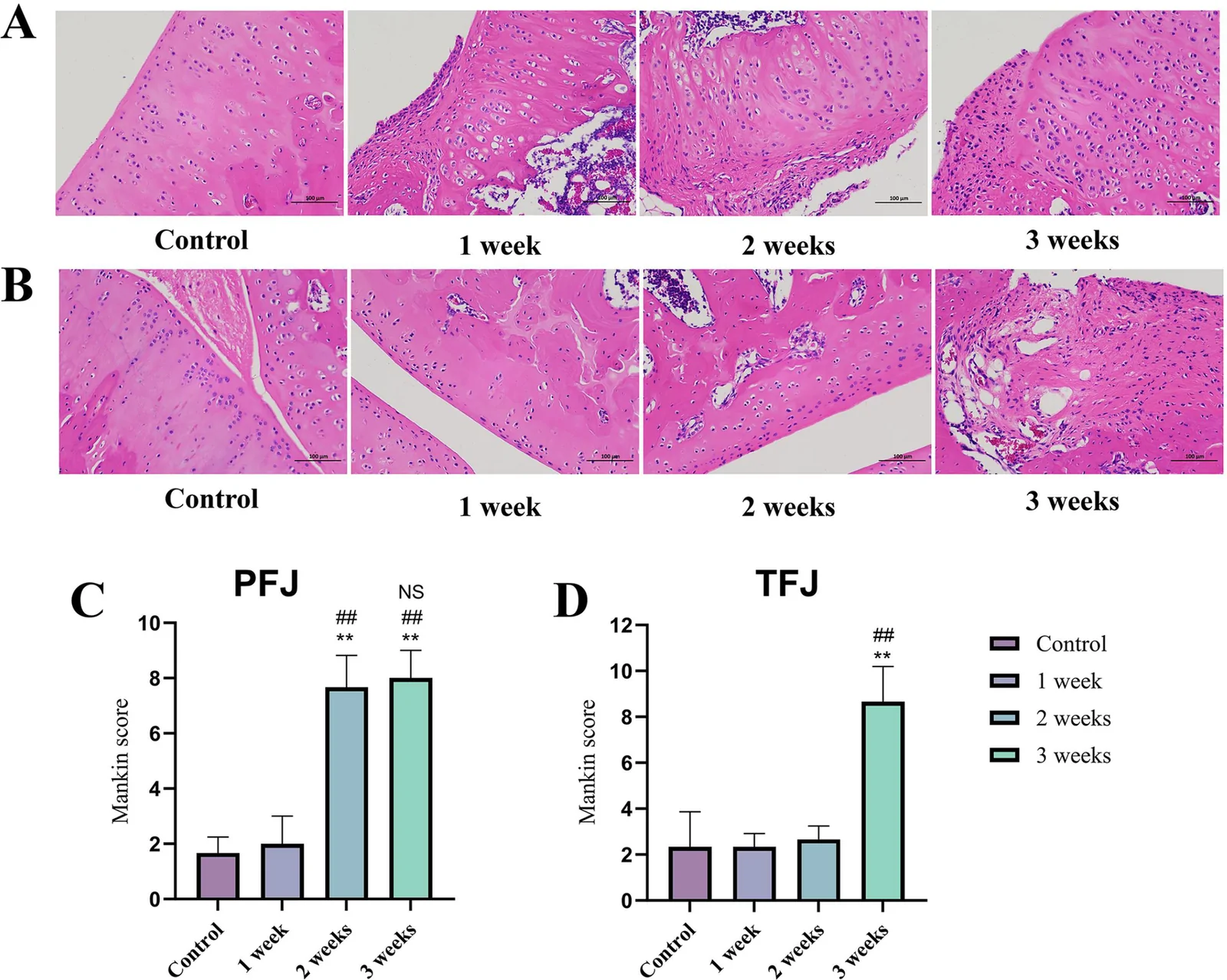
Histological changes and mankin scores in the PFJ and TFJ. **(A,B)** Representative H&E-stained sections of PFJ and TFJ cartilage from the control and 1-, 2-, and 3-week model groups. **(A)** PFJ sections showing the articular cartilage of the patella and femoral trochlea. **(B)** TFJ sections showing the articular cartilage of the femoral condyle and tibial plateau. **(C,D)** Mankin scores of PFJ and TFJ cartilage, respectively. **P < 0.01 versus the control group; ##*P* < 0.01 versus the 1-week model group; NS, *P* > 0.05, not significant. Scale bar, 100 μm.

H&E staining showed intact cartilage surfaces in the PFJ and TFJ of the control group, with clear zonal architecture and regularly arranged chondrocytes. At 1 week after surgery, the PFJ cartilage displayed mild surface irregularity and localized roughness, accompanied by disorganization of the chondrocytes. At 2 weeks after surgery, the cartilage surface became more irregular, the zonal architecture was further disrupted, and focal degenerative areas had increased in number. These pathological changes progressed further by postoperative week 3. In contrast, TFJ cartilage exhibited relatively mild changes at postoperative weeks 1 and 2, whereas more pronounced structural damage observed at week 3 (Figures 1A, B).

Mankin scores of the PFJ gradually increased with postoperative time. Specifically, the scores in the 2- and 3-week model groups were significantly higher than those in the control group and 1-week model group. In the TFJ, Mankin scores showed no significant differences in the 1- and 2-week groups but showed a marked increase in the 3-week group. These findings were consistent with the H&E staining results, indicating that in this model, cartilage degeneration occurred earlier and was more severe in the PFJ than in the TFJ (Figures 1C, D).

### Progressive loss of proteoglycans in the patellofemoral joint

**Figure 2:**
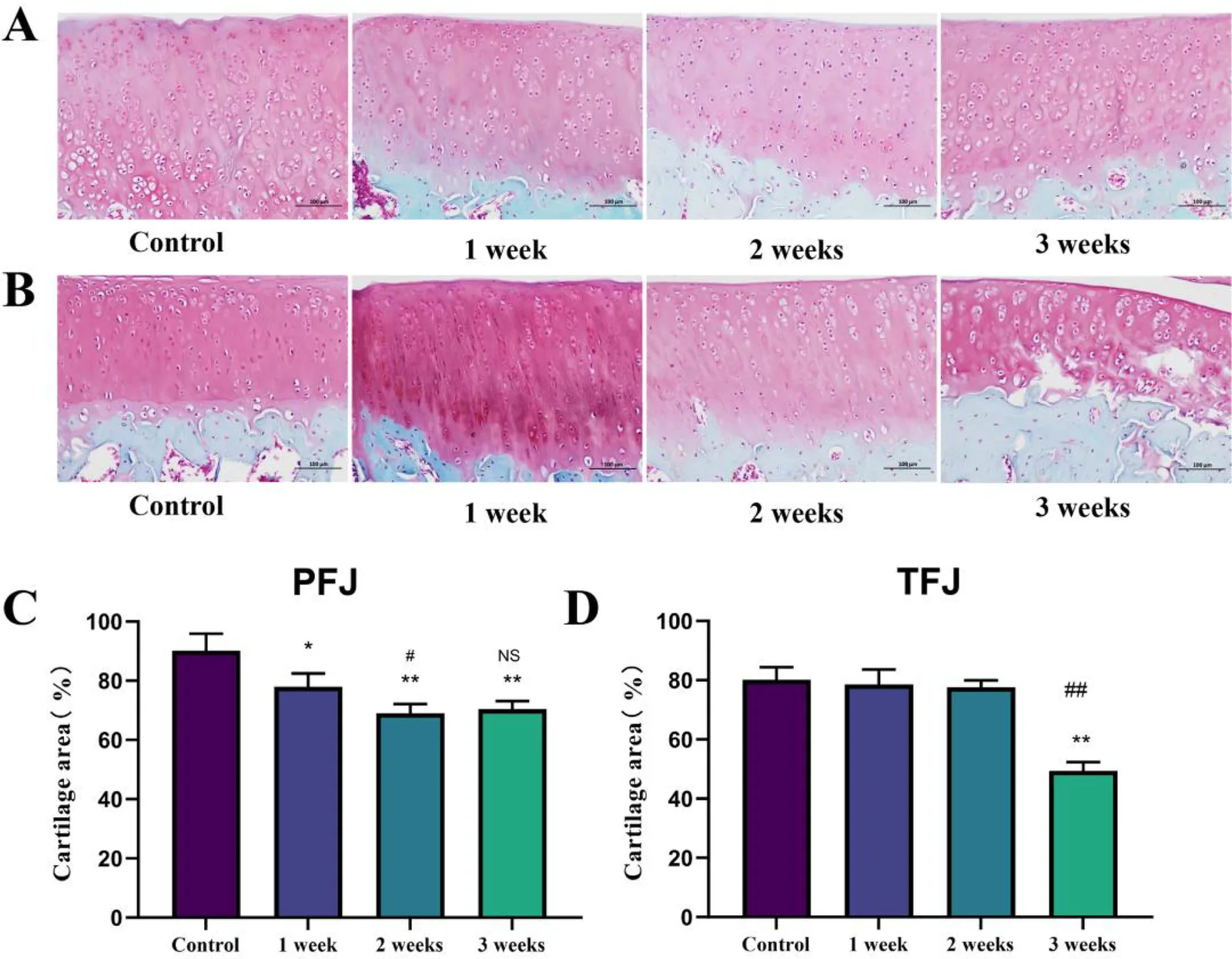
Safranin O–fast green staining and quantitative analysis of proteoglycan loss in the PFJ and TFJ. **(A,B)** Representative Safranin O–Fast Green-stained sections of PFJ and TFJ cartilage from the control and 1-, 2-, and 3-week model groups. **(A)** PFJ sections showing the articular cartilage of the patella and femoral trochlea. **(B)** TFJ sections showing the articular cartilage of the femoral condyle and tibial plateau. **(C,D)** Percentages of the Safranin O-positive area in PFJ and TFJ cartilage, respectively. *P < 0.05 and **P < 0.01 versus the control group; #*P* < 0.05 and ##*P* < 0.01 versus the 1-week model group; NS, *P* > 0.05, not significant. Scale bar, 100 μm.

Safranin O-Fast green staining showed intense and uniform red staining of the cartilage matrix in the PFJ and TFJ of the control group, indicating abundant proteoglycan content. At 1 week after model, Safranin O staining of the PFJ cartilage was already reduced, as indicated by localized fading and a decrease red-stained area. At 2 weeks after model induction, the staining intensity and red-stained area decreased further. These changes persisted at postoperative week 3. In contrast, the reduction in safranin O staining in the TFJ cartilage was comparatively delayed. No obvious changes were observed at postoperative weeks 1 or 2, whereas a marked reduction in staining was observed at week 3 (Figures 2A, B).

Quantitative analysis of the percentage of the Safranin O-positive area revealed a significant decrease in PFJ cartilage at postoperative week1, with further reductions at weeks 2 and 3. In contrast, a significant reduction in the percentage of the Safranin O-positive area in the TFJ cartilage was observed only in the 3-week model group. No significant differences were observed among the control, 1-week and 2-week groups (Figures 2C, D).

### The PFOA model accelerates glycosaminoglycan loss in the PFJ cartilage

**Figure 3:**
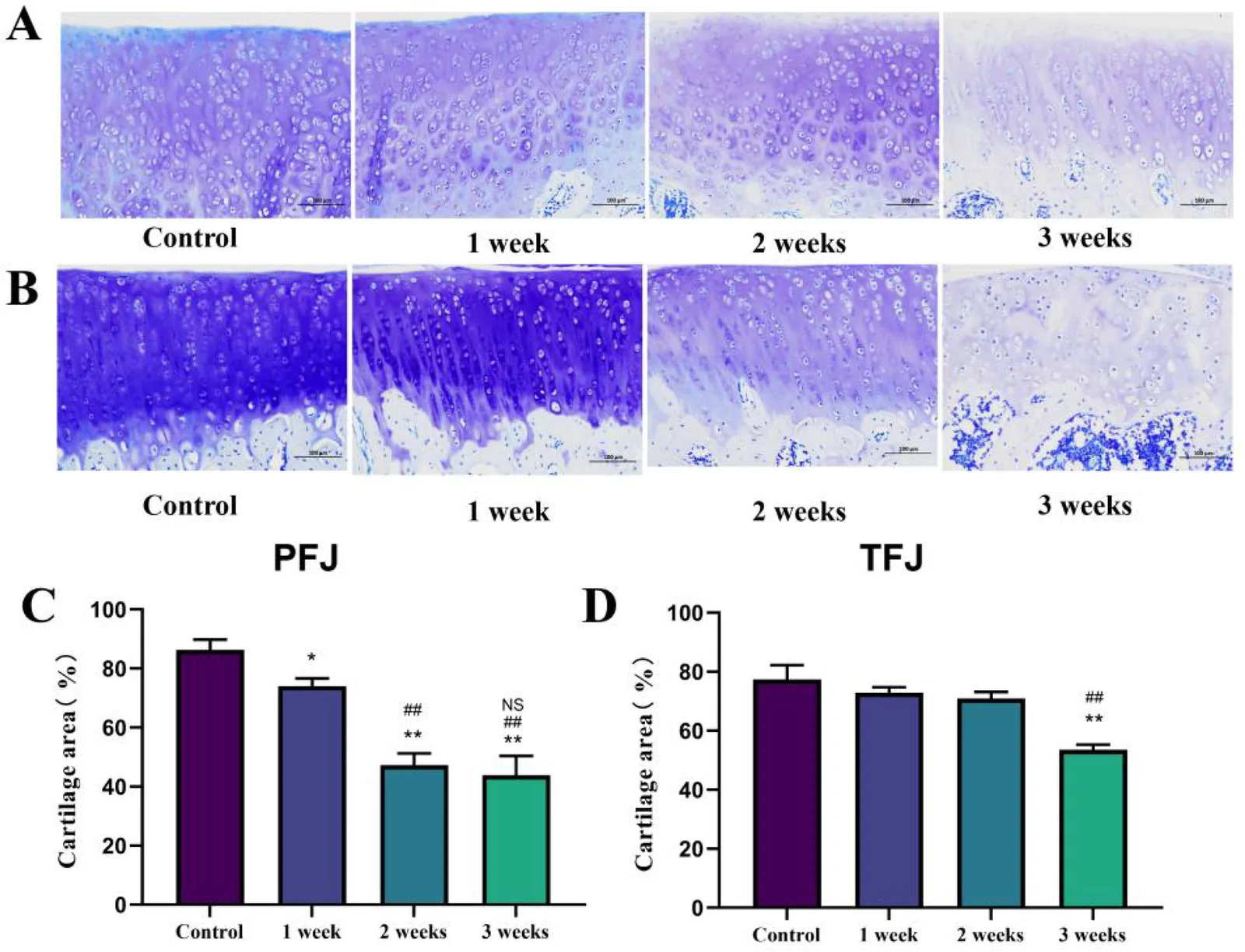
Toluidine blue staining and quantitative analysis of glycosaminoglycan loss in the PFJ and TFJ. **(A,B)** Representative toluidine blue-stained sections of PFJ and TFJ cartilage from the control and 1-, 2-, and 3-week model groups. **(A)** PFJ sections showing the articular cartilage of the patella and femoral trochlea. **(B)** TFJ sections showing the articular cartilage of the femoral condyle and tibial plateau. **(C,D)** Percentages of the toluidine blue-positive area in PFJ and TFJ cartilage, respectively. *P < 0.05 and **P < 0.01 versus the control group; ##*P* < 0.01 versus the 1-week model group; NS, *P* > 0.05, not significant. Scale bar, 100 μm.

Toluidine blue staining showed that, in the control group, the cartilage matrix of both the PFJ and TFJ was uniformly and deeply stained, indicating an intact distribution of GAGs. After model induction, staining in the PFJ cartilage progressively weakened over time, indicating a gradual decrease in GAG content. By contrast, comparable changes in the TFJ occurred later and were less severe (Figures 3A, B). Quantitative analysis of the toluidine blue-positive area confirmed the histological observations. The PFJ cartilage showed an earlier reduction in the percentage of the positivly stained area than the TFJ, with the latter showing a more pronounced decline only in the later postoperative period (Figures 3C, D).

### Cartilage metabolic imbalance in synovial fluid and serum

To assess early changes in cartilage metabolism after model induction, ELISA was used to measure PIICP, MMP-13, CTX-II, and COMP levels in the synovial fluid and serum of rats in each group. At 1 week after model induction, PIICP levels in both synovial fluid and serum were significantly lower than those in the control group, whereas MMP-13, CTX-II, and COMP levels were significantly higher (all *P* < 0.01), indicating reduced type II collagen anabolism and increased cartilage matrix catabolism at an early stage.

**Figure 4:**
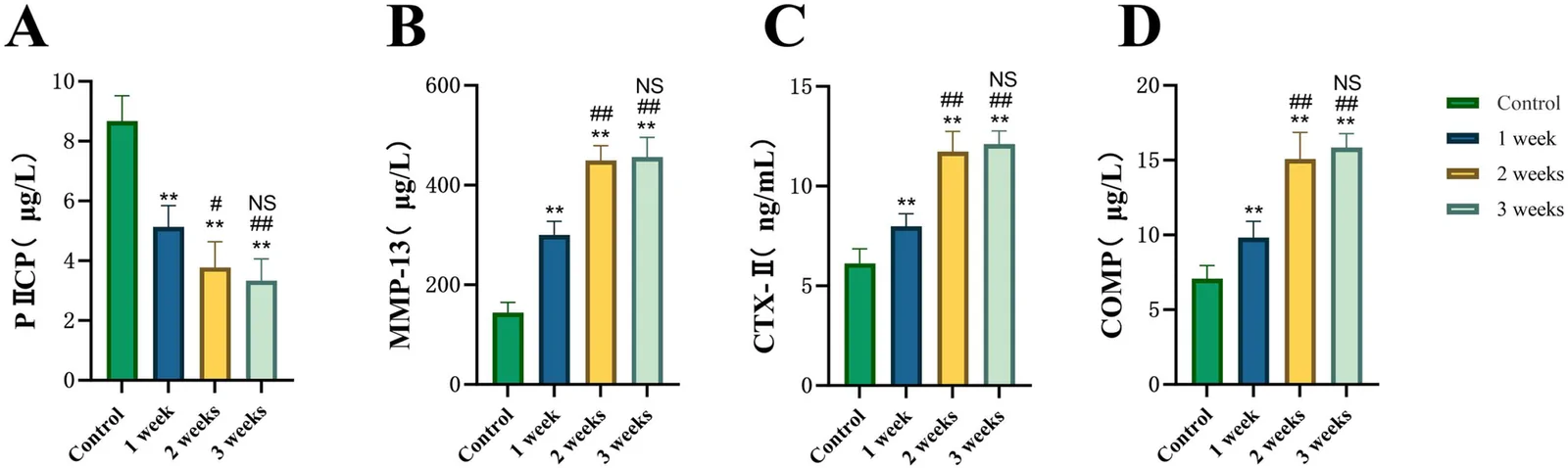
Quantitative analysis of cartilage metabolic markers in synovial fluid. **(A-D)** Concentrations of PIICP, MMP-13, CTX-II, and COMP in synovial fluid, respectively. **P < 0.01 versus the control group; #P < 0.05 and ##*P* < 0.01 versus the 1-week model group; NS, *P* > 0.05, not significant between the 2- and 3-week model groups.

**Figure 5:**
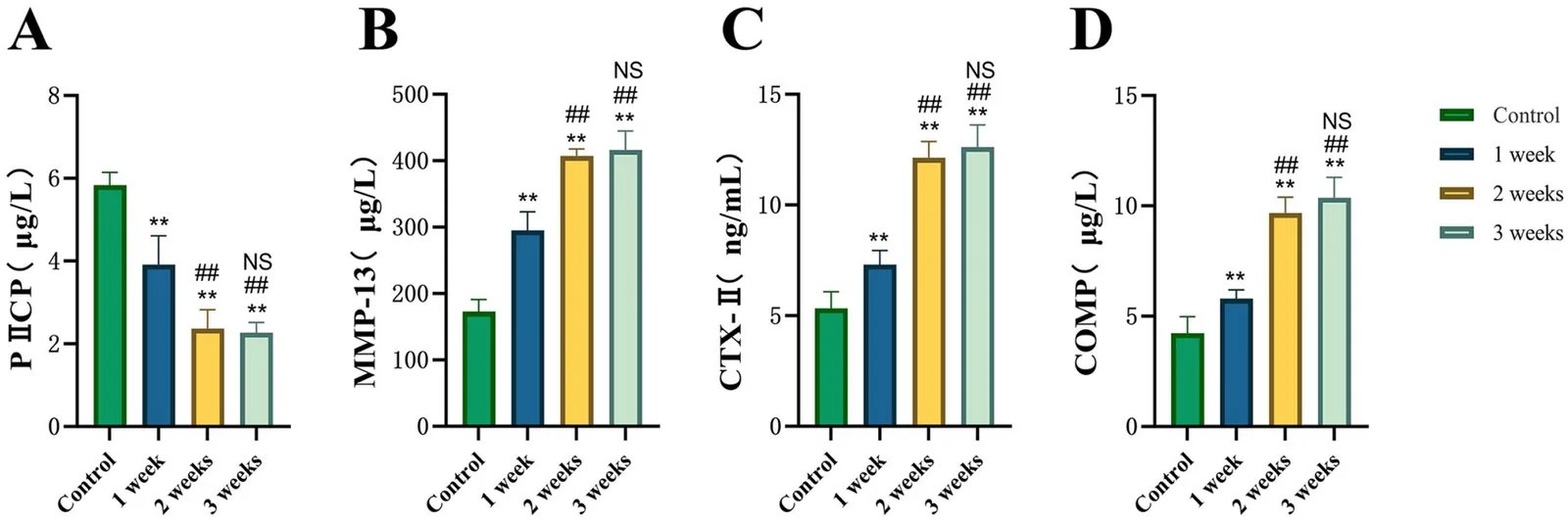
Quantitative analysis of cartilage metabolic markers in serum. **(A-D)** Concentrations of PIICP, MMP-13, CTX-II, and COMP in serum, respectively. **P < 0.01 versus the control group; ##*P* < 0.01 versus the 1-week model group; NS, *P* > 0.05, not significant between the 2- and 3-week model groups.

Compared with the 1-week model group, these changes were more pronounced in the 2-week and 3-week model groups. PIICP levels continued to decrease, whereas MMP-13, CTX-II, and COMP continued to increase. Further analysis showed that PIICP levels in synovial fluid were significantly lower in the 2-week model group than in the 1-week model group (*P* < 0.05), and this decrease was more pronounced in the 3-week model group (*P* < 0.01). Serum PIICP levels were also significantly lower in the 2-week and 3-week model groups than in the 1-week model group (both *P* < 0.01). In both synovial fluid and serum, MMP-13, CTX-II, and COMP levels were further elevated in the 2-week and 3-week model groups compared with the 1-week model group (all *P* < 0.01). However, no significant differences were observed between the 2-week and 3-week model groups for any marker (all *P* > 0.05) (Figures 4, 5).

Moreover, trends in each marker in synovial fluid and serum were generally consistent, indicating that the metabolic changes in this model were reflected not only in the local joint environment but also, to some extent, in peripheral blood. Combined with the histological observations and quantitative analysis described above, these findings indicate that the model induced decreased cartilage anabolism and increased catabolism during the early postoperative period, accompanied by histological degeneration characterized by predominant involvement of the PFJ. These data further support the successful establishment of the model.

## Discussion

This study established an early-stage degenerative model with predominant involvement of the PFJ within a relatively short period. This phenotype was induced by transecting the medial one-third of the patellar ligament and the medial patellar retinaculum. Compared with the TFJ, the PFJ showed earlier cartilage surface irregularity, cellular disorganization, and reduced matrix staining. These changes were accompanied by decreased levels of cartilage synthesis-related markers and elevated levels of degradation-related markers in both synovial fluid and serum. Taken together, these findings suggest that this model was not intended to replicate generalized KOA in the conventional sense. Rather, it represents an early-stage, localized, mechanically driven PFOA phenotype predominantly involving the PFJ. This model may provide useful insights into early cartilage injury associated with patellar instability. The 1-, 2-, and 3-week time points were selected to capture the early degenerative response after the induction of patellar instability. Therefore, the present study mainly demonstrates the feasibility of rapidly inducing PFJ-predominant early degeneration, rather than fully characterizing the long-term progression of PFOA.

Previous studies using patella baja and patellar instability models have shown that changes in patellar position or tracking alone can induce substantial degeneration of patellofemoral cartilage. These findings suggest that localized mechanical abnormalities may representimportant initiating factors in PFOA (16, 19). PFOA is not merely a consequence of focal cartilage wear. Increasing evidence indicates that PFOA is closely associated with abnormal patellar tracking, malalignment, and localized mechanical overload (6, 25). Patellar instability, lower limb malalignment, and chronic overload can alter the contact area and pressure distribution within the PFJ, leading to cartilage injury and degeneration (26). The medial stabilizing structures are essential for limiting lateral patellar displacement, maintaining normal tracking during early flexion, and stabilizing patellofemoral contact (27). Once medial restraint is weakened, the patella may become more prone to lateral translation, lateral tilt, or subluxation during flexion and extension. These alterations can result in abnormal patella motion within the femoral trochlea and aberrant local contact stress distribution (28). Therefore, in this study, the medial patellar supporting structures were weakened, rather than directly damaging the articular cartilage, to simulate patellar kinematic abnormalities and local mechanical imbalance after attenuation of the medial stabilizers of the PFJ (19). Using this model, we observed that histological degeneration appeared earlier in the PFJ than in the TFJ. This finding further supports the hypothesis that patellar instability-induced mechanical imbalance contributes to early PFJ cartilage damage.

Compared with the various models described above, the present model has a more clearly defined lesion origin and compartmental bias. Classic meniscal instability-induced OA models, including DMM and MMT, are well established and reproducible approaches for studying KOA. However, these models primarily disturb meniscal stability and tibiofemoral load transmission, and the resulting lesions are mainly located in the tibiofemoral weight-bearing compartment (29). Therefore, although DMM and MMT models are valuable for studying post-traumatic or instability-related knee OA, they are not specifically designed to reproduce PFJ-centered patellar maltracking or local mechanical imbalance. Intra-articular injection of monosodium iodoacetate (MIA) can also induce cartilage degeneration and pain-related behaviors within a short period. However, its primary mechanism involves chemical inhibition of chondrocyte metabolism and is therefore more suitable for studying chemically induced cartilage destruction (20). The patella baja model, by contrast, alters PFJ contact mechanics by changing patellar height, but it usually requires a more complex surgical procedure (16).

Building on the previously reported patellar instability-induced model (19), the present study further emphasizes surgical standardization and early compartment-specific validation. Specifically, transection of the medial one-third of the patellar ligament and medial patellar retinaculum was used to weaken medial patellar stability in a defined anatomical manner, while interval suturing and fixation of the lateral peripatellar soft tissues helped maintain the patella in a mildly lateralized position. This design was intended to reduce variability in patellar maltracking, avoid complete dislocation or direct cartilage injury, and preserve the major stabilizing structures of the TFJ as much as possible. Together with the earlier and more pronounced histological and matrix changes observed in the PFJ than in the TFJ, these findings suggest that the present model is more suitable for studying early-stage, mechanically driven PFOA with preferential PFJ involvement, rather than generalized whole-knee OA or advanced diffuse degeneration.

In this study, H&E staining and Mankin scoring initially confirmed the histological validity of the model through assessment of cartilage structure. The PFJ showed surface irregularity, focal roughening, and disorganized chondrocyte arrangement during the early postoperative period, while changes in the TFJ were relatively delayed. These findings suggest that the model does not induce synchronous, diffuse damage throughout the entire knee joint. Instead, it preferentially triggers early structural degeneration in the PFJ. Proteoglycans are essential components that maintain cartilage water retention and compressive resistance, and their loss is considered an important feature of early cartilage matrix degeneration in osteoarthritis (30). Reduced glycosaminoglycan content reflects impaired cartilage matrix homeostasis and may weaken the load-bearing and mechanical buffering capacity of cartilage (31). Safranin O-Fast Green and Toluidine Blue staining further showed that proteoglycan and glycosaminoglycan loss occurred earlier and was more pronounced in PFJ cartilage than in TFJ. These results further support the view that matrix-level degeneration occurrs earlier in the PFJ and is accompanied by early structural changes. Moreover, these alterations progressively worsened over time.

Analysis of soluble biomarkers provided a metabolic complement to the histological findings. In osteoarthritis, disruption of extracellular matrix (ECM) homeostasis represents a critical early pathogenic event: anabolic responses are suppressed, whereas catabolic processes are upregulated, ultimately leading to the progressive loss of proteoglycans, glycosaminoglycans, and the type II collagen network (32). Previous reviews have emphasized that markers such as MMP-13, CTX-II, and COMP reflect, at different levels, the processes of cartilage ECM degradation and matrix remodeling (33). A decrease in PIICP suggests inhibition of type II collagen synthesis (34), whereas elevated MMP-13, CTX-II, and COMP indicate enhanced matrix degradation and aggravated cartilage damage (35–37). Prior studies have demonstrated that CTX-II is closely associated with cartilage degradation, COMP is linked to structural damage and disease activity, and MMP-13 is regarded as one of the most important type II collagen-cleaving enzymes in osteoarthritis (38). In the present study, decreased PIICP levels, together with increased levels of MMP-13, CTX-II, and COMP, suggested that ECM metabolic imbalance had already occurred during the early stage after model induction, rather than being confined to end-stage tissue destruction. The trends observed in synovial fluid and serum were generally consistent, indicating that local joint degeneration may be partly reflected in peripheral biomarkers.

Notably, most soluble biomarkers reached a statistical plateau between weeks 2 and 3, whereas histological degeneration continued to worsen. This divergence may reflect the different biological meanings of soluble biomarkers and histological structural assessment. MMP-13, CTX-II, and COMP mainly reflect the current activity of cartilage matrix turnover, degradation, and remodeling, whereas histological changes represent the cumulative burden of structural damage within cartilage tissue. Therefore, the plateau of humoral biomarkers between weeks 2 and 3 does not necessarily indicate cessation of cartilage degeneration. Instead, it may suggest that the initial acute mechanical and inflammatory response had reached a relatively stable metabolic phase, while cumulative structural arthropathy continued to progress under persistent abnormal patellofemoral loading. This finding supports the view that synovial fluid and serum biomarkers may be more sensitive to active cartilage turnover rate than to accumulated structural damage. Similar temporal patterns have been noted in recent osteoarthritis studies, suggesting that osteoarthritic pathology exhibits time-dependent characteristics (39). However, because the observation period was limited to 3 weeks, longer follow-up with later time points is needed to determine whether cartilage degeneration continues to progress, stabilizes, or transitions toward more advanced pathological changes.

This study has several limitations. First, the degree of lateral patellar displacement was assessed mainly by intraoperative observation, without quantitative imaging-based measurements. Second, the follow-up period was limited to 3 weeks after surgery, and later time points such as 4 and 6 weeks were not included in the original experimental design. Therefore, although the current results support the establishment of an early-stage PFOA model, they are insufficient to fully determine whether degeneration continues to progress, stabilizes, or transitions toward more advanced pathological changes over time. Future studies should extend the observation period by including additional time points, such as 4 and 6 weeks, to more comprehensively characterize the temporal evolution of this model. In addition, changes in the synovium, infrapatellar fat pad, and subchondral bone were not systematically evaluated, and gait or joint mechanics were not further analyzed. Together, these factors may affect the overall assessment of the pathological completeness and biomechanical validity of this model.

## Conclusion

This study established and preliminarily validated a rat PFOA model induced by patellar instability. The model induced cartilage structural damage, disrupted ECM homeostasis, and caused metabolic abnormalities within a relatively short period, with preferential involvement of the PFJ. Future studies should extend the observation period, incorporate imaging, biomechanical, and molecular mechanistic analyses, and conduct a more systematic evaluation of the model. Such work may support the application of this model in experimental research on PFJ-targeted therapeutic interventions.

## Data Availability

The raw data supporting the conclusions of this article will be made available by the authors, without undue reservation.
